# Supplementary material for: Impact of dominance rank specification in dyadic interaction models
Source: PLoS One. 2023 Jul 20;18(7):e0277130. doi: 10.1371/journal.pone.0277130 (PMC10358901; doi:10.1371/journal.pone.0277130)
Supplement: S1 File — (DOCX) [file pone.0277130.s002.docx]

**Supplementary Material: Impact of dominance rank specification in dyadic interaction models**

Mielke, Alexander ^1,2,3^

1 School of Psychology and Neuroscience, University of St Andrews, UK

2 Primate Models for Behavioural Evolution Lab, School of Anthropology and Museum Ethnography, Oxford, UK

3 Taï Chimpanzee Project, Centre Suisse de Recherches Scientifiques en Côte d'Ivoire, Abidjan, Côte d'Ivoire

**Abstract**

Dominance rank is a vital descriptor of social dynamics in animal societies and regularly used in studies to explain observed interaction patterns. However, researchers can choose between different indices and standardizations, and can specify dyadic rank relations differently when studying interaction distributions. These researcher degrees of freedom potentially introduce biases into studies and reduce replicability. Here, I demonstrate the impact of researcher choices by comparing the performance of different combinations of rank index, standardization, and model specification when explaining dyadic interaction patterns in sooty mangabeys (*Cercocebus atys atys*). I show that while no combination consistently performed best across interaction types (aggression, grooming, proximity, supplants), model specifications allowing for nonlinear patterns performed better than other models on average. Choices made in pre-processing and model building impacted model performance and subsequent interpretation of results. Researchers could end up describing social systems differently based on the same data. These results highlight the impact of researcher choices in the processing of behavioural data and potential limitations when using indirect species comparisons in animal behaviour research. To increase repeatability, researchers could make the impact of their processing choices more transparent and report results using a variety of indices and model specifications.

**Model Graphs**

All graphs were build based on the nonlinear interaction models using Elo ratings that were standardised between 0 and 1 and then z-standardised – thus, higher values indicate a more powerful individual.

*Model 1: Aggression*

Best Model: Higher/Lower Factor


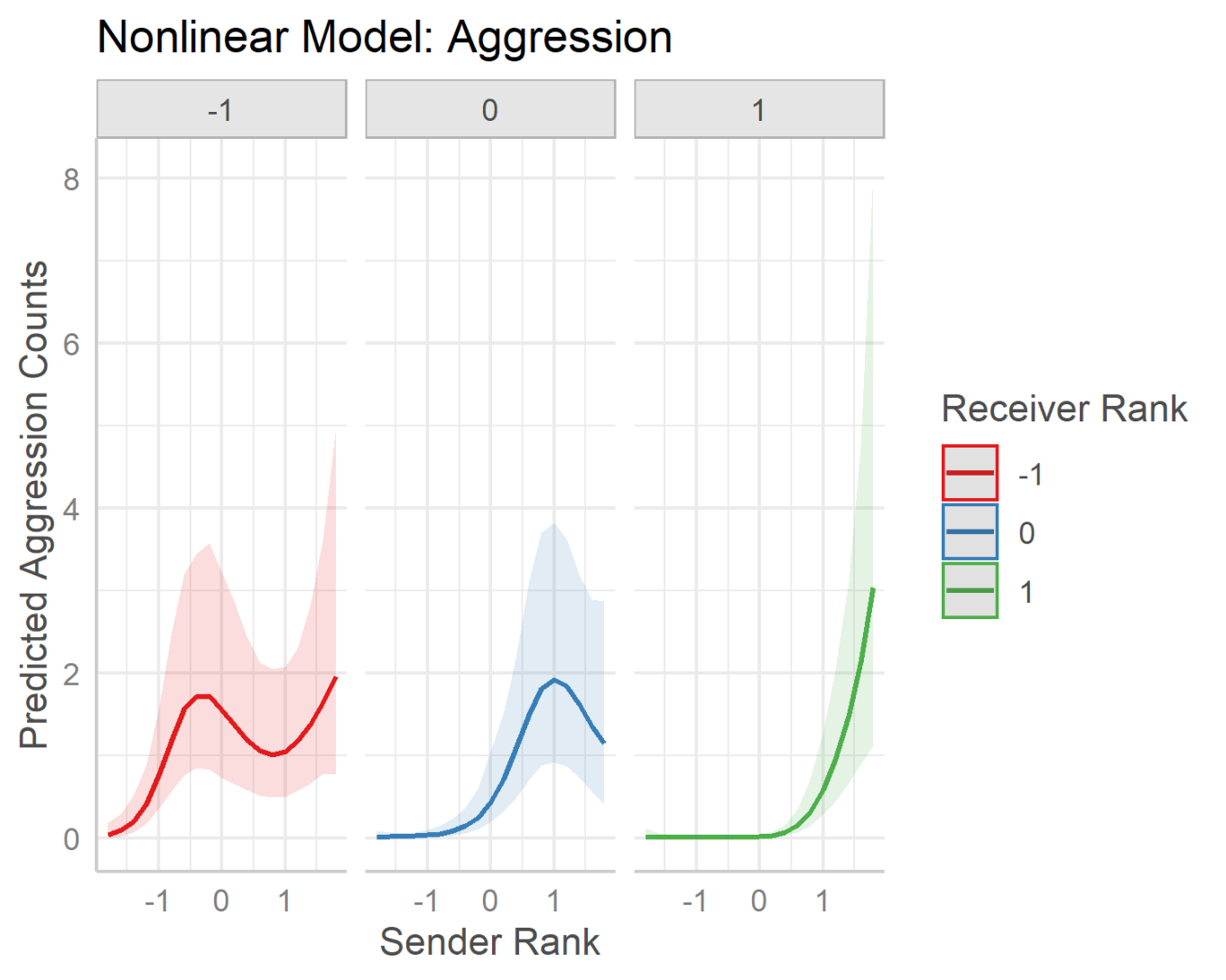


Figure S1: Predicted aggression events per year, based on the Sender Rank (x-axis, z-standardised) and the Receiver Rank (z-standardised, cut into three). The left graph shows aggression towards low-ranking victims, the middle graph towards medium-ranked victims, the right graph towards high-ranked victims.

*Model 2: Grooming*

Best Model: Nonlinear Interaction, David’s Score, Proportional Standardisation


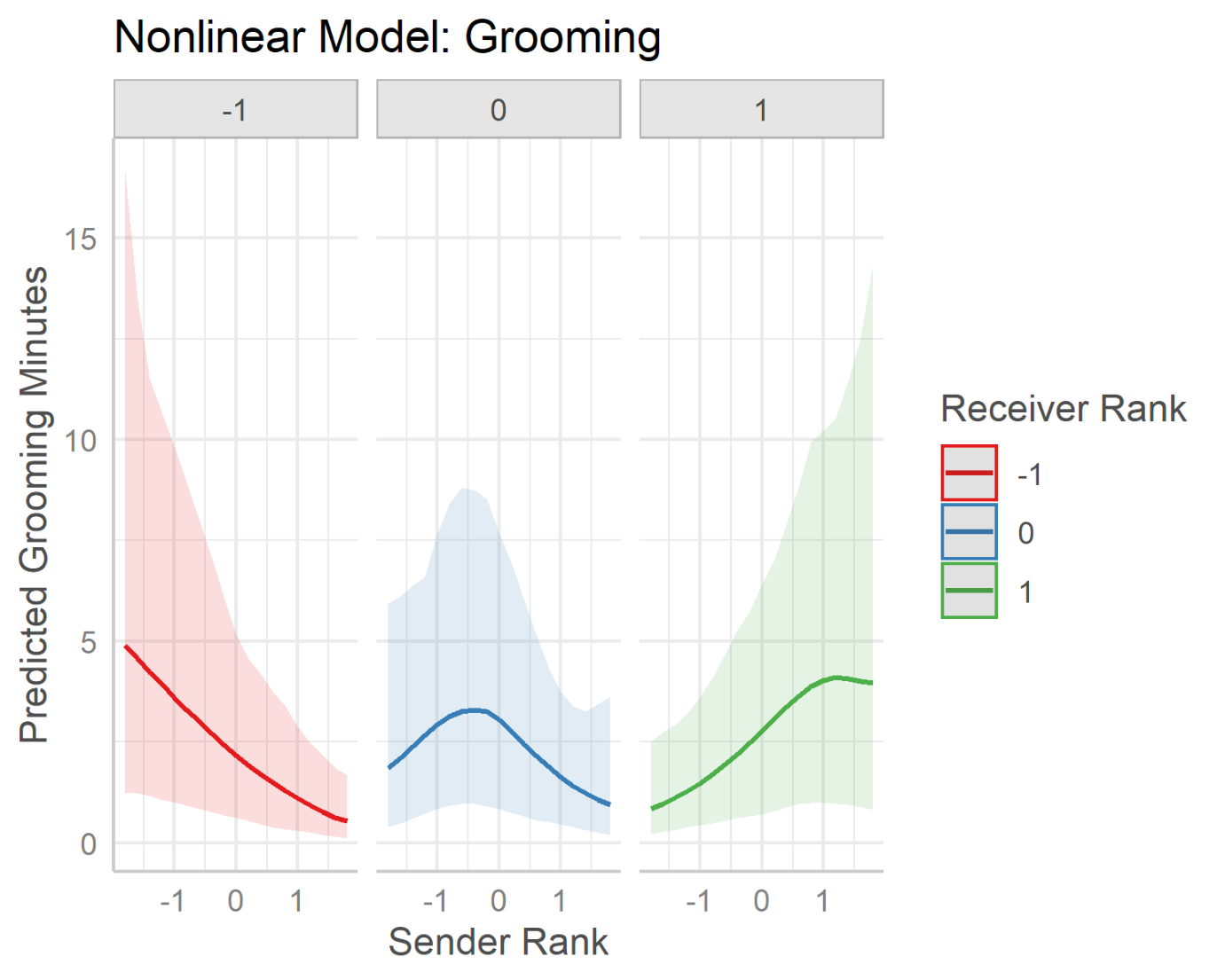


Figure S2: Predicted grooming in minutes per year, based on the Sender Rank (x-axis, z-standardised) and the Receiver Rank (z-standardised, cut into three). The left graph shows grooming towards low-ranking partners, the middle graph towards medium-ranked partners, the right graph towards high-ranked partners.

*Model 3: Proximity*

Best Model: Nonlinear Interaction, Elo rating, Proportional Standardisation


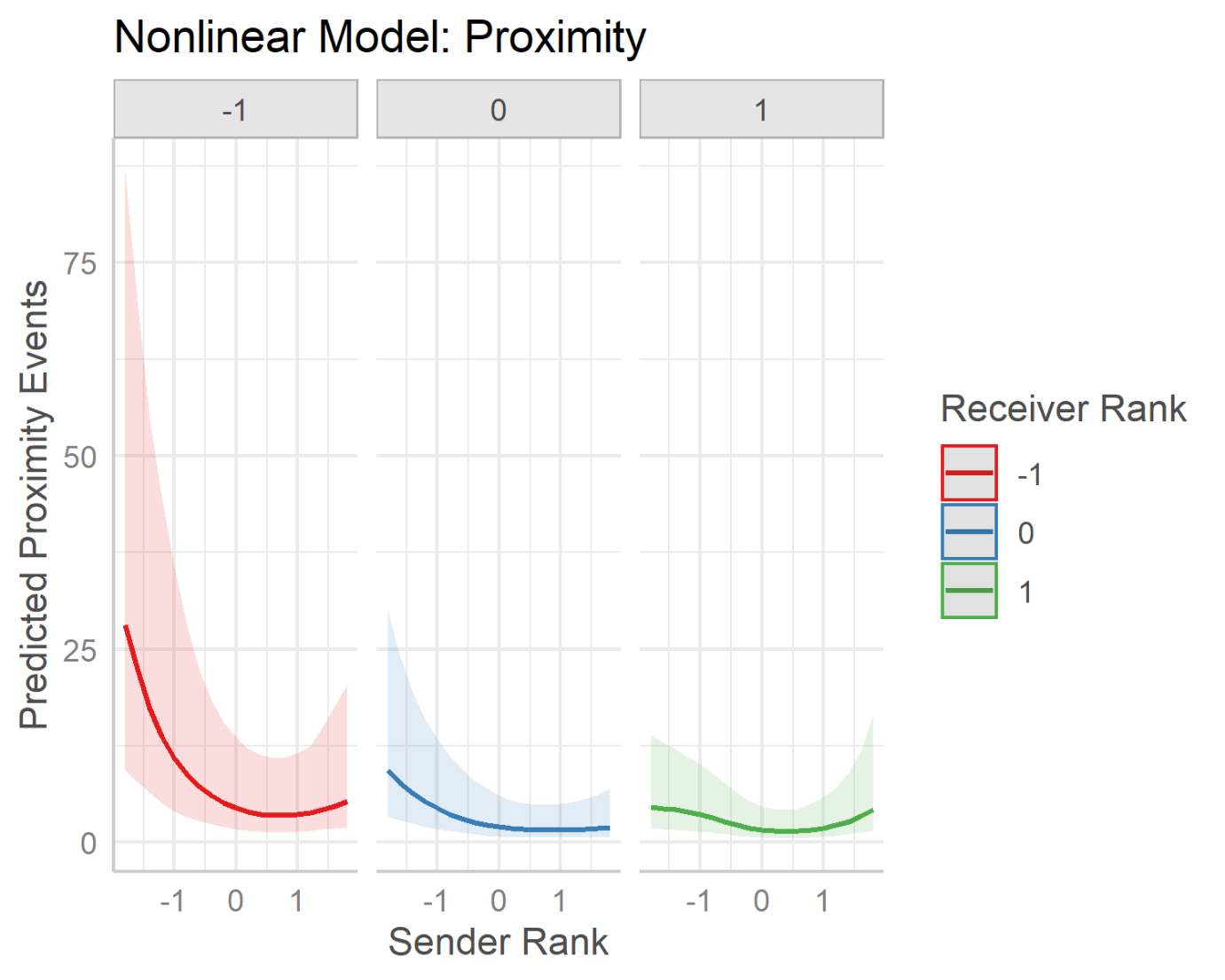


Figure S3: Predicted proximity events per year, based on the Sender Rank (x-axis, z-standardised) and the Receiver Rank (z-standardised, cut into three). The left graph shows proximity with low-ranking partners, the middle graph with medium-ranked partners, the right graph with high-ranked partners.

*Model 4: Supplants*

Best Model: Higher/Lower Factor


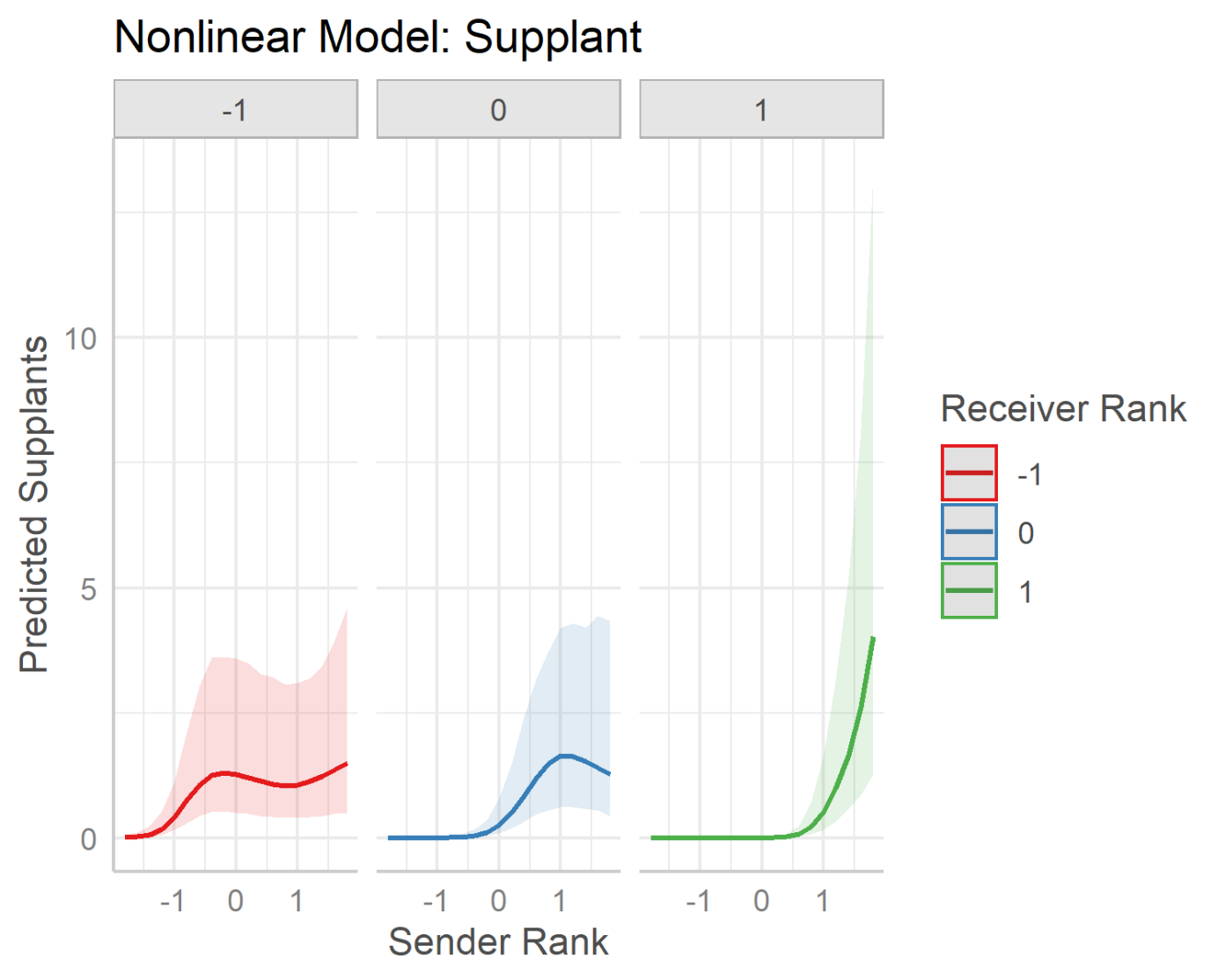


Figure S1: Predicted supplant events per year, based on the Sender Rank (x-axis, z-standardised) and the Receiver Rank (z-standardised, cut into three). The left graph shows supplants of low-ranking victims, the middle graph of medium-ranked victims, the right graph of high-ranked victims.
