# Supplementary material for: Impact of dominance rank specification in dyadic interaction models
Source: PLoS One. 2023 Jul 20;18(7):e0277130. doi: 10.1371/journal.pone.0277130 (PMC10358901; doi:10.1371/journal.pone.0277130)
Supplement: Attachment — Submitted filename: Review Comments to the Author.docx [file pone.0277130.s004.docx]

**Review Comments to the Author**

**Reviewer #3**

It is shown that assuming equidistance among dominance ranks is a better predictor than calculating raw differences in the values of the dominance indices, and I find it very interesting. The author argues that “there is no indication that the numeric distances produce [sic] by some human-made algorithms are meaningful for how animals structure their dominance hierarchies, and raw values will be influenced to a larger degree by measurement error” (lines 423-424), which is argued to be supported by the results and discussed in great detail. However, the discussion would benefit from warranting some space to elaborate on the possible implications if such finding proved to be correct also for other species and systems, and thus potentially a general characteristic of social systems. For example, there are multiple cases in which such numerical distances are assumed to be meaningful in the literature, with implications on interpretation of results (which is a relevant topic for this paper). For example, the methodology proposed by de Vries and colleagues (2006) uses the difference in David’s score values among individuals as a basis to calculate hierarchical steepness, and it’s a widely used metrics with over 300 citations. In addition, this result may be dependent on the characteristics of this study species and of female-female interactions specifically (e.g., high linearity, possibly high steepness), although the author correctly restricts the statement to these analyses: “equidistance is the assumption that minimises error in this case,” lines 425-426. If this result were consistently replicated in multiple species with different characteristics, it would have interesting implications. I suggest the author expand the discussion on this point to allow also non specialist readers to understand the possible implications of this finding alongside future avenues of research. Also, as a small typo I think the author intended to use “produced” and not “produce” in the sentence (line 423). –

*I thank the reviewer for this valuable observation! I have added this further information for readers to clarify this:*

*‘It is worth remembering that raw Elo ratings and David’s Scores are relative measures of winning likelihoods, which implies that the lower-ranking individual could, in principle, win the contest. While this may be true for aggression in several animal species, it is not true for many behaviours that are used to create dominance hierarchies in primates, which are strictly one-directional, transitive and are acknowledgements of the lower-ranking individual of the higher-ranking individual’s position (such as displacements/supplants or pant grunts; Mielke et al. 2019). This has implications on common indices that use raw David’s Scores to test hierarchy steepness (de Vries et al. 2006) and assume that the differences between individuals are meaningful. However, it is worth investigating whether the effect presented here holds across species and whether it would be replicated when using contest competition to create hierarchies.’ (L.423)*
